# Subarachnoid Hemorrhage Revealing a Cervical Intradural Extramedullary Cavernous Malformation: A Case Report and Literature Review

**DOI:** 10.7759/cureus.90832

**Published:** 2025-08-23

**Authors:** Karim Baayoud, Nour el Houda Laffinti, Kadiri A Naji, Zakariae Benyaich, Mohamed Lmejjati

**Affiliations:** 1 Neurosurgery, University Hospital Souss Massa, Agadir, MAR; 2 Neurological Surgery, University Hospital Souss Massa, Agadir, MAR

**Keywords:** acute subarachnoid hemorrhage, cavernous malformation, intradural extramedullary cavernoma, spinal cord cavernoma, spinal cord cavernous malformation, spinal mri

## Abstract

Spinal cavernomas are uncommon among spinal vascular malformations. Most arise in the vertebrae, with some extending into the extradural space. A few are intramedullary, while intradural extramedullary cases are even rarer. This study reports a newly diagnosed case of a cervical intradural extramedullary cavernous malformation (IDEM) at the University Hospital Center of Agadir. A 48-year-old female patient presented with severe headaches of abrupt onset. A brain CT scan showed a diffuse subarachnoid hemorrhage in the basal cisterns, with extension into the ventricles. Following this finding, the patient was admitted to the neurosurgery department for further investigation and developed a sudden-onset right hemiparesis. A cerebral MRI scan showed ventricular flooding with triventricular hydrocephalus. Subsequently, a spinal cord MRI was performed, which identified an extramedullary intradural lesion at the C3/C4 level, characterized by a heterogeneous signal, consistent with an IDEM cavernoma. The patient underwent ventriculoperitoneal shunting for hydrocephalus, followed by surgical resection of the lesion. Histopathology confirmed a cavernous malformation. Postoperatively, the patient achieved full motor recovery, with no recurrence at the one-year follow-up.

## Introduction

Cavernous malformations are low-flow vascular lesions of the central nervous system. Spinal cavernomas comprise approximately 5% of all cavernomas and are more commonly found within the extradural or intramedullary compartments [1]. Intradural extramedullary cavernous malformation (IDEM) cavernomas represent a scarce variant, originating from the inner surface of the dura mater, the pial surface of the spinal cord, or the vasculature of the spinal nerves. The clinical manifestations of these lesions can vary considerably. Given the relative rarity of these lesions, their diagnosis and management present significant challenges. This article offers an overview of a rare case involving a cervical IDEM cavernous malformation that resulted in spontaneous subarachnoid hemorrhage (SAH) in a 48-year-old patient, diagnosed at the University Hospital Center of Agadir. The discussion encompasses a comprehensive review of the clinical, radiological, pathological, and therapeutic aspects of this rare case, as well as an examination of the recent literature on the subject.

## Case presentation

A 48-year-old woman, with no prior medical history, presented with a sudden onset of severe headaches (Visual Analog Scale (VAS) score = 8/10) exhibiting partial response to standard analgesic treatment. This clinical presentation was accompanied by episodes of vomiting (three occurrences). Notably, there were no signs of limb weakness, seizures, diminished visual acuity, or altered consciousness. The symptoms manifested in the absence of fever, with the patient maintaining a stable general condition.

The initial clinical evaluation revealed a patient devoid of sensory-motor deficits, exhibiting normal muscle tone, intact osteotendinous reflexes, and no impairment of cranial nerves or higher cognitive functions (grade 1 according to the World Federation of Neurosurgical Societies scale). The remainder of the examination yielded unremarkable findings, apart from the presence of nuchal rigidity.

**Figure 1:**
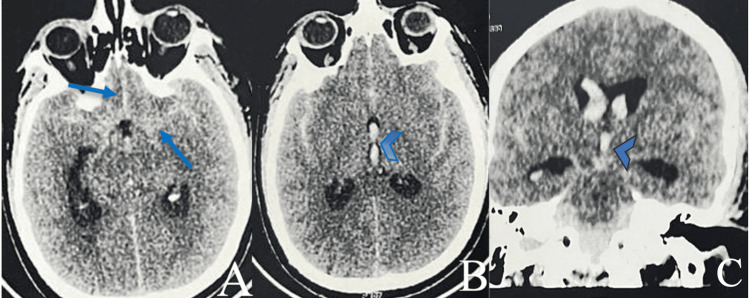
Brain CT scan: axial plane (A, B) without contrast and coronal plane (C) post-contrast injection. (A) Diffuse subarachnoid hemorrhage of the basal cistern (arrow) with (B, C) ventricular flooding into the third ventricle (arrowhead) (grade 4 on the modified Fisher scale).

A non-contrast head CT revealed diffuse SAH in the basal cisterns, with ventricular flooding and acute hydrocephalus, corresponding to a grade 4 on the modified Fisher scale (Figure 1).

**Figure 2:**
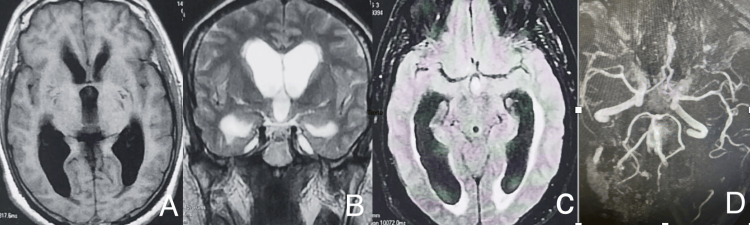
Brain MRI: (A) T1-weighted image axial plane, (B) T2-weighted image coronal plane, (C) fluid-attenuated inversion recovery sequence axial plane, and (D) MR angiography sequences. (A-C) Subarachnoid hemorrhage with intraventricular flooding in the third ventricle associated with an acute moderate active triventricular hydrocephalus. (D) MR angiography sequences showing no abnormalities in the circle of Willis.

A few hours later, the patient developed sudden-onset right limb weakness. The clinical examination revealed right hemiparesis (3/5), with no pyramidal syndrome. A brain MRI was performed, showing hypersignal in fluid-attenuated inversion recovery (FLAIR) imaging, suggestive of SAH associated with mild intensity active triventricular hydrocephalus, with normal angiographic sequences (Figure 2).

The patient underwent an emergency ventriculoperitoneal shunting for her hydrocephalus, with partial regression of headaches (VAS score = 6/10) and persistence of her right hemiparesis.

Considering the presence of the right hemiparesis and the clear evidence of SAH on imaging, coupled with the absence of visible ischemic lesions or vasospasm on the brain MRI sequences, we determined it was essential to perform a cervical MRI to ensure a comprehensive assessment.

**Figure 3:**
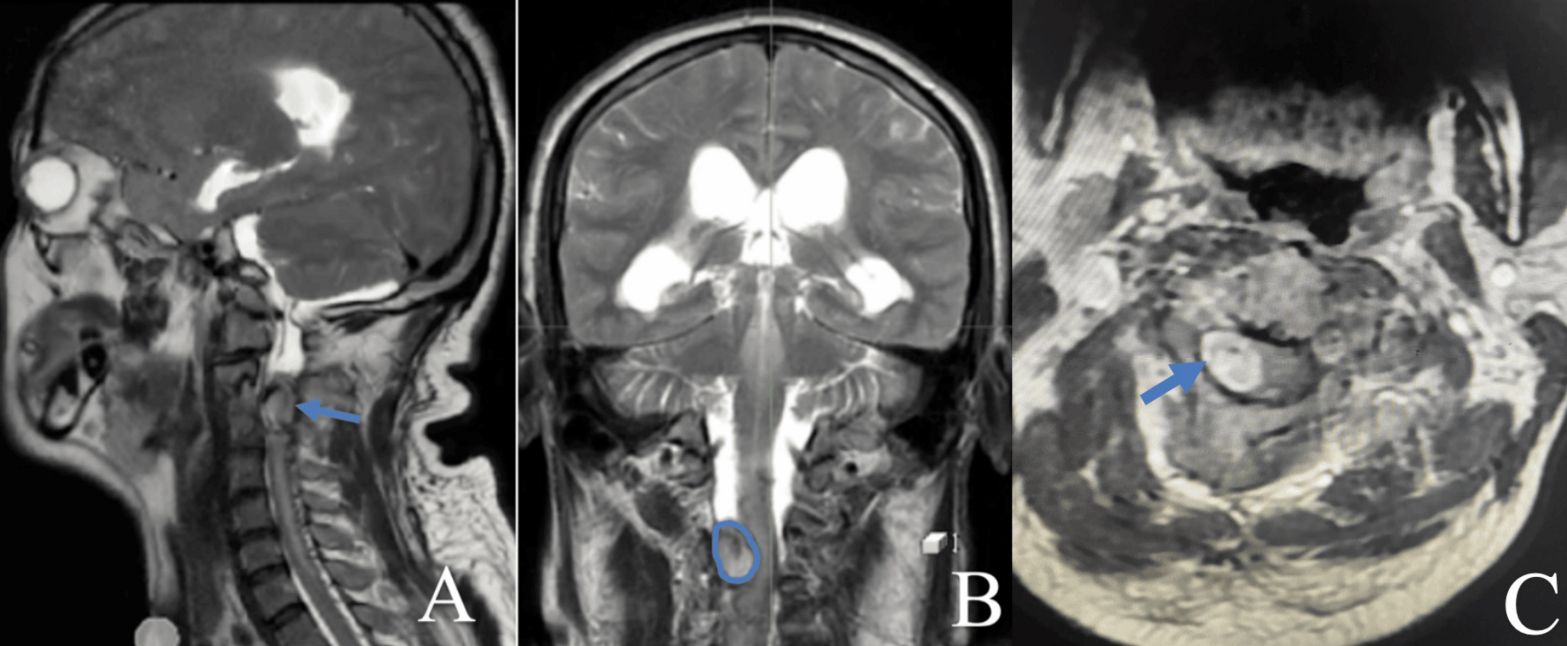
Cervical MRI: T2-weighted images, sagittal (A), coronal (B) and axial (C) planes. Right-sided cervical extramedullary lesion at the C3-C4 level (arrow, highlighted in blue) appears heterogeneous with mixed signal on T2 (hypersignal associated with a central area of hypointensity consistent with hemosiderin deposition). This lesion is exerting a mass effect on the cervical spinal cord that appears displaced to the left.

The spinal MRI identified a right-sided cervical extramedullary lesion at the C3-C4 level. This lesion appeared hypointense on T1-weighted images and hyperintense on T2-weighted images (Figure 3), with a hypointense peripheral ring (hemosiderin ring) suggesting a cavernous malformation. The relationship between this lesion and the dura was difficult to assess. This lesion exerted a mass effect on the cervical spinal cord.

**Figure 4:**
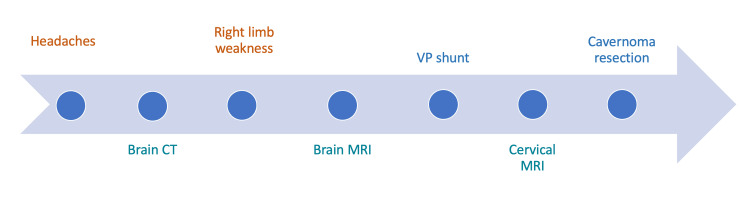
A summary of the clinical evolution, key diagnostics, and interventions in this case.

The patient underwent surgical resection of the lesion using a posterior approach. After performing a C3/C4 laminotomy and opening the dura, we observed a lesion that originated from the dura, was lateralized to the right, and was exerting a mass effect on the cervical spinal cord. We proceeded with the complete removal of the lesion. The following timeline summarizes the patient's course of events within our department (Figure 4).

**Figure 5:**
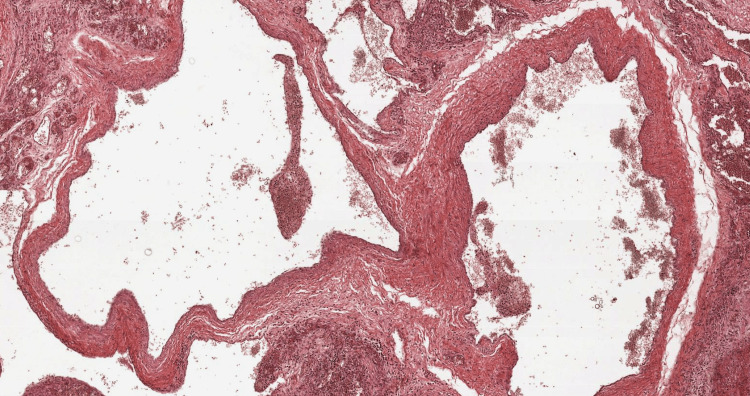
Microphotograph showing dilated vascular spaces lined by flattened endothelium. The lumina contains red blood cells (hematoxylin and eosin, 100×).

Histological examination confirmed the diagnosis of a cavernous malformation (Figure 5).

**Figure 6:**
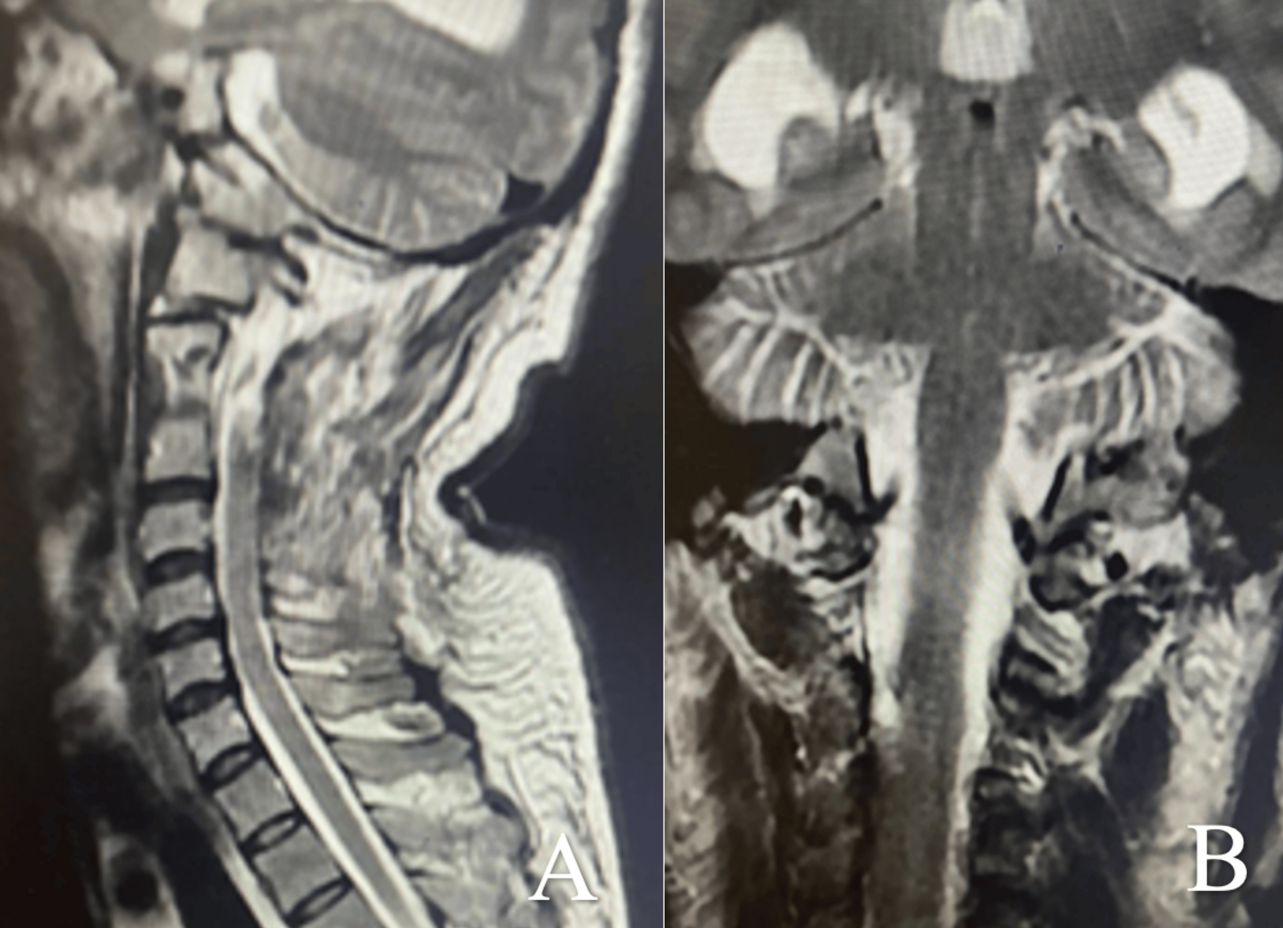
Cervical MRI: postoperative T2-weighted images, sagittal (A) and coronal (B) planes. Postoperative imaging confirmed the total resection of the intradural extramedullary cavernous malformation cavernoma.

The postoperative course was favorable, with complete motor recovery following rehabilitation. Imaging confirmed total resection of the lesion (Figure 6). The one-year follow-up showed no recurrence.

## Discussion

Spinal cavernous malformations are uncommon, varying between 5-16% of spinal vascular malformations, with intradural extramedullary locations being the least common subtype. Most cavernous malformations originate in the vertebrae. Only 3% are intramedullary, and intradural-extramedullary cases are even less common [2]. Approximately 75 cases of intradural extramedullary cavernous malformations have been reported, including this case [3-6]. Only 12 cases, including our case, have been described as being located at the cervical level [6-15] (Table 1). Intradural extramedullary cavernous malformations are mostly located at the thoracolumbar level [4,5,10,14,16].

**Table 1:** A Summary of all cervical intradural extramedullary cases described in the literature. CCJ = craniocervical junction; CSF = cerebrospinal fluid; ND = not described; SAH = subarachnoid hemorrhage

Author and year	Age (years), sex	Level	Origin	Symptoms	Lumbar puncture	Surgery extent	Outcome
Ortner et al., 1973 [6]	22, Male	C4-C7	ND	SAH, tetraplegia, cranial nerve failure	Bloody CSF	Total	No improvement
Acciarri et al., 1992 [7]	54, Female	C2–C3	Dura	SAH	Bloody CSF	Total	Complete
Harrison et al., 1995 [8]	37, Male	CCJ-C5	Root	Brown-Sequard syndrome	ND	Total	Incomplete
Nozaki et al., 2003 [9]	51, Male	C5–C6	Root	Shoulder pain, sensorimotor deficit	ND	Total	Complete
Park et al., 2003 [10]	61, Male	C1–C2	ND	Headache	Bloody CSF	Total	Complete
Rachinger et al., 2006 [5]	56, Male	C6–C7	Root	Shoulder pain, total spine pain, sensory deficit	ND	Total	Complete
Kivelev et al., 2008 [11]	44, Male	C5–C6	Root	Brown-Sequard syndrome, incontinence	ND	Total	Incomplete
Henderson et al., 2018 [12]	65, Female	C6-C7	Root	Radiculopathy, sensory deficit	ND	Total	Complete
Vicenty et al., 2019 [13]	76, Female	C7–8	Root	Neck pain	ND	Total	Complete
Frank et al., 2022 [14]	45, Male	C5–C6	Root	SAH, transient radiculopathy	Bloody CSF	Total	Complete
Gader et al., 2024 [15]	58, Female	C2-C4	Pia-mater	Occipital and cervicobrachial neuralgia	ND	Total	Complete
Our case	48, Female	C3-C4	Dura	SAH, right hemiparesis	ND	Total	Complete

Only two cases of intradural extramedullary cavernous malformations have been described, compressing the S1 nerve root [17,18]. The age of the patients ranged between the second and seventh decades of life, and cases predominantly occurred in men [14].

In the literature, we found that the clinical expression of IDEM cavernomas varies depending on the stage of the disease and the location within the spinal canal. Only four cases have been described as being revealed by an SAH (Table 1).

On MRI, the presentation of these lesions varies, with a predominantly mixed signal intensity in T1- and T2-weighted images due to blood products of varying ages. The contrast enhancement is variable [19]. The lesion can be circumscribed by a hypointense border on MRI, indicating hemosiderin deposition, which strongly suggests cavernous malformations [20].

However, diagnosis can be rather challenging in some cases, where the radiological appearance may resemble certain tumor lesions [21,22]. Minimal enhancement in the context of cavernous lesions can be utilized to differentiate them from tumoral lesions (meningioma or schwannoma), which show more pronounced enhancement.

McQueen et al. provided several recommendations regarding the management of intradural extramedullary. Expectant management does not offer symptom improvement and risks further deterioration and complications associated with SAH [3]. Endovascular treatments have higher rates of recurrence and progression of myelopathy following embolization [13]. Surgery is the treatment of choice for symptomatic lesions, especially when hemorrhage is involved, and often results in good outcomes, as demonstrated here and in other reports [3]. In 66 cases out of 75, the resection was complete. When described precisely during a surgical procedure, cavernomas were mostly adherent to nerve roots. Root sacrifice may be necessary, and a total resection was possible in all cases, with a complete recovery in seven cases [14]. Even in the cases with root sacrifice, the outcome was still favorable.

## Conclusions

Spinal cavernomas are rare, and IDEM cavernomas are the least common type. To date, only 12 cases, including ours, have been reported in the cervical region. This case contributes to the limited literature on spinal cavernous malformations and reinforces the need for a systematic diagnostic approach in similar scenarios. Intradural extramedullary cavernomas are an unusual cause of SAH. A spinal MRI should be considered if cerebral imaging results are negative during the SAH evaluation. Early diagnosis and surgical intervention are crucial for preventing recurrence and maintaining neurological function.
